# Biomarkers in Pneumonia—Beyond Procalcitonin

**DOI:** 10.3390/ijms20082004

**Published:** 2019-04-24

**Authors:** Meropi Karakioulaki, Daiana Stolz

**Affiliations:** 1 School of Medicine, Aristotle University of Thessaloniki, 54124 Thessaloniki, Greece; merop.i@hotmail.com; 2 Clinic of Pulmonary Medicine and Respiratory Cell Research, University Hospital, Petersgraben 4, CH-4031 Basel, Switzerland

**Keywords:** biomarkers, procalcitonin, pneumonia, novel

## Abstract

Pneumonia is the leading infectious cause of mortality worldwide and one of the most common lower respiratory tract infections that is contributing significantly to the burden of antibiotic consumption. Due to the complexity of its pathophysiology, it is widely accepted that clinical diagnosis and prognosis are inadequate for the accurate assessment of the severity of the disease. The most challenging task for a physician is the risk stratification of patients with community-acquired pneumonia. Herein, early diagnosis is essential in order to reduce hospitalization and mortality. Procalcitonin and C-reactive protein remain the most widely used biomarkers, while interleukin 6 has been of particular interest in the literature. However, none of them appear to be ideal, and the search for novel biomarkers that will most sufficiently predict the severity and treatment response in pneumonia has lately intensified. Although our insight has significantly increased over the last years, a translational approach with the application of genomics, metabolomics, microbiomics, and proteomics is required to better understand the disease. In this review, we discuss this rapidly evolving area and summarize the application of novel biomarkers that appear to be promising for the accurate diagnosis and risk stratification of pneumonia.

## 1. Pneumonia

Increasing antimicrobial resistance is a life-threatening worldwide phenomenon. Lower respiratory tract infections are one of the leading causes for antibiotic consumption and thus for the emergence of resistant microbial strains [1].

Despite the rapid development of new treatments, pneumonia continues to cause a high rate of health complications and death [2]. It has been reported that in 2010 there were 120 million episodes of pneumonia in children younger than five years old, 14 million of which progressed to severe episodes [3]. Thus, pneumonia is now considered to be the leading infectious cause of mortality worldwide [4] and one of the most common infections contributing significantly to the burden of antibiotic consumption [1]. It is classified as either community-acquired or nosocomial, according to the environment from which the patient contracted the infection.

Community-acquired pneumonia (CAP) is the third most common cause of death globally, the major cause of death and sepsis in developed countries, and accounts for between 5% and 12% of all cases of adult lower respiratory tract infections (LRTIs) managed by primary care physicians [5,6,7,8]. It is one of the most common infectious diseases, with significant mortality and frequent need for intensive care support due to the occurrence of respiratory insufficiency and multiorgan involvement [9]. Especially among the elderly and among patients with premorbid conditions, CAP is associated with a high risk of mortality, and approximately 10% of patients hospitalized require admission to an intensive care unit (ICU) [10,11,12]. Additionally, CAP has a high rate of reoccurrence as a significant proportion of patients discharged from the hospital are readmitted within 30 days [13]. The incidence of CAP requiring hospitalization is 20.6 per 10,000 cases each year [14]. The most challenging task for a physician is the risk stratification of patients with CAP, and early diagnosis is essential in order to reduce hospitalization and mortality [15].

Nosocomial pneumonia is commonly divided into two distinct groups: hospital-acquired pneumonia (HAP) and ventilator-associated pneumonia (VAP). HAP denotes an episode of pneumonia that occurs more than 48 h after hospital admission and was not in incubation at the time of the admission [16]. VAP is defined as pneumonia that occurs more than 48 h after the initiation of invasive mechanical ventilation [16].

VAP is a life-threatening complication in the ICU and is associated with longer duration of mechanical ventilation, longer hospital stay, increased treatment costs, and increased attributable mortality [17]. One of the most challenging problems in VAP is the lack of a “gold standard” method of diagnosis [18]. The commonly used criteria are based on clinical variables that lack specificity [19]; as a result, up to 50% of patients diagnosed with VAP do not really have this condition (over-diagnosis) [20]. Consequently, there is a great need for a clinical tool that will improve the precise diagnosis and the decision-making process in order to avoid over-diagnosis (and thus antibiotic overuse) or under-diagnosis and late diagnosis, both of which may result in worse outcomes [16].

## 2. Impact of Incorrect Use of Antibiotics

Most cases of pneumonia result from bacterial infection, which is treated with antibiotics. However, some noninfectious causes, such as pulmonary embolism, malignancy, and congestive heart failure may also lead to symptoms resembling CAP. In addition, viral pneumonia is a well-described entity, particularly among immunocompromised patients [21]. In such cases, the erroneous diagnosis is usually suspected only after failure of the antibiotic treatment, and the life-threatening risks associated with these untreated nonbacterial causes increase rapidly [22,23].

Undeniably, under-prescription of antibiotics may lead to an increased risk of development of respiratory failure and sepsis. It has been shown that a delay of more than 4 h in administering antibiotics to a patient with CAP after hospital admission is significantly associated with increased mortality rates [24]. On the other hand, over-prescription of antibiotics may include risks such as unnecessarily exposing patients to the side effects of antibiotics without achieving a more rapid recovery; increased probability for development of antibiotic-resistant microorganisms; and an increase in patient recovery time, costs, and workload [25,26,27,28,29]. Alarmingly, the WHO published its first global report on microbial resistance in 2014, warning that we are heading for the post-antibiotic era, where common infections may no longer be treatable by the available antibiotics [1].

## 3. The Role of Biomarkers

A biomarker is any molecule, structure, or process that can be measured in the body or its excretions and influences or predicts the incidence of a disease [30]. Biomarkers are seen as an effective way of monitoring a patient’s response to infection by speculating on the disease severity and response to treatment [31]. This also allows earlier and better identification of patients with severe life-threatening infections and supports the selection of a more appropriate treatment method [16].

An ideal diagnostic biomarker for infection should be low or absent when infection is absent and high in the presence of a specific infection. It should, ideally, provide results sooner than conventional culture reports [17]. Furthermore, an ideal biomarker should be of either high specificity or sensitivity and useful for characterizing severity and monitoring response to treatment even in the absence of clinical signs [32,33]. Preferably, it should not be expensive or invasive, should be timely, and should help avoid excess antibiotic use [16].

Biomarkers in pneumonia may be the ones that indicate inflammation or may be released specifically after lung injury due to infection [17]. The measured levels of biomarkers should be interpreted cautiously and always be correlated with clinical findings as many confounding factors should be taken into consideration for interpretation [31]. Factors like age, antibiotic pretreatment, chronic hepatic disease, corticosteroids, renal impairment, and viral confection can critically affect some biomarker levels and thus their sensitivity and specificity regarding treatment failure and clinical stability [34,35,36,37,38]. Hence, results should be interpreted in line with the clinical presentation, and they should never substitute clinical judgment [31].

## 4. Beyond Procalcitonin

The search for the ideal biomarker for pneumonia is ongoing, and multiple molecules are undergoing rigorous investigation [31]. C-reactive protein (CRP) and procalcitonin (PCT) remain the most robust and widely used biomarkers, while interleukin 6 (IL-6) has been of particular interest research-wise [39,40,41,42]. PCT is a 116 amino acid prohormone of calcitonin [32]. In the presence of bacterial infection, the *CALC-1* gene is upregulated, producing PCT in large amounts by the macrophage and monocytic cells throughout the body, especially in the liver, lung, and intestine [43]. The rise of PCT is immediate due to its cytokine-like behavior [31]. It is identifiable within 2–3 h, with a peak at 6 h [32]. However, PCT has a number of limitations. It is elevated in a variety of noninfectious conditions, such as cirrhosis, pancreatitis, mesenteric infraction, burns, and aspiration pneumonitis [44,45,46]. Additionally, its diagnostic and predictive value declines in patients with severe sepsis and in localized infections (e.g., endocarditis, empyema) [44,47,48]. Studies differ as to what are the appropriate negative cut-off points for PCT [44,49].

Overall, more than 7000 patients have so far been included in randomized, controlled studies comparing a PCT-guided approach to the standard use of antibiotics in lower respiratory tract infections [50]. The vast majority of these studies depicted a statistically significant and clinically relevant decrease in antibiotic exposure [51]. Specifically, in CAP, PCT has been shown to significantly reduce the initiation and duration of antibiotic therapy. In addition, the use of PCT has led to either similar or better clinical outcomes, with some studies suggesting a decrease in mortality by 5% at 28 days and 7.4% at one year [52].

However, PCT appears to be less reliable for the diagnosis of VAP, especially compared to cases presenting with infections acquired at the community level. The reason for this difference is probably that patients with VAP or HAP have already developed systemic inflammation response syndromes, multiple organ failure, and/or previous infection. All these conditions raise PCT levels and thus make the usual cut-off points proposed for the diagnosis of less severe infection less useful. Therefore, a decrease in the initial levels instead of fixed cut-off levels should be used to define the point in which antibiotic therapy can be suspended [53]. A recent trial also highlighted the need for adherence to the PCT guidance protocol in order to decrease antibiotic use [54].

Additionally, PCT appears to be the best performing diagnostic marker for the detection of pneumococcal pneumonia in pediatric patients and could lead to early beta lactam therapy [55,56]. However, the use of PCT should be considered with caution in pediatric patients as it is still unknown whether there is an advantage in severe and complicated CAP, and further studies are needed to better understand its role in such conditions [56]. Moreover, neonates demonstrate elevations in PCT levels in the first three days of life [57].

Thus, although PCT gives much more sensitive and specific information than previous systemic biomarkers for infection, its drawbacks have inspired and motivated more rigorous research for the further discovery of novel biomarkers for pneumonia.

## 5. C-reactive Protein (CRP)

CRP synthesis is rapidly upregulated in the liver in response to cytokines originating at the site of pathology (particularly IL-6, which induces CRP mRNA) [16,32,40]. Thus, CRP levels determine its rate of synthesis in the liver, and this rate indicates the response to the inflammation intensity [40,58]. Therefore, CRP is a superior biomarker for more complex acute-phase characteristics, e.g., leukocytosis and fever [40]. CRP secretion begins in 4–6 h and peaks at 36–50 h, potentially limiting its efficacy in predicting early treatment failure [40,59]. Criticism of the role of CRP in diagnostics includes not only the delay in response to clinical stimulus but also its poor specificity as it is elevated in a variety of pathologies, such as trauma, surgery, burns, and immunological-mediated inflammatory diseases [31]. Mendez et al. successfully demonstrated that CRP is a significant independent predictor for the absence of severe complications in CAP [60]. Another large prospective study of 570 patients in a major Scottish hospital demonstrated that CRP <100 mg/L on admission was significantly associated with reduced 30-day mortality, need for mechanical ventilation, and/or inotropic support and complicated pneumonia [61].

The use of single measurements of CRP in the diagnosis of VAP has not consistently shown positive results [16]. A single elevated plasma CRP concentration is not very informative; consequently, CRP is not specific enough to diagnose nosocomial pneumonia [62]. However, the constant monitoring of CRP levels appears to be useful in the early prediction of VAP and the response to antibiotics [63,64,65].

Because CRP is produced in the liver, some conditions are associated with artificially lower levels, such cirrhosis and hypoproteinemia [66]. In addition, immunosuppressed patients, in particular those receiving oral steroids, tend to have no increase in CRP, even in severe bacterial infection [67]. Another challenge seems to be the differentiation of inflammation and infection using CRP [68].

## 6. IL-6

IL-6 is involved in a variety of hematopoietic, immune, and inflammatory responses as it enhances T-cell differentiation through the induction of IL-2 [31]. One big advantage of IL-6 over CRP and PCT is an immediate response to infection [44,69]. Another diagnostic advantage of IL-6 over PCT is that IL-6 is a more sensitive biomarker of localized infection (e.g., effusions) [44]. Various studies have demonstrated that IL-6 might be a useful predictor of treatment failure and mortality. In this respect, it has been reported that elevated IL-6 indicates a higher risk of 30-day mortality (84% sensitivity and 87% specificity) [39]. Moreover, IL-6 levels have been shown to have a good correlation with various clinical severity scores (pneumonia severity index [PSI], CURB 65, and MEWS) [39]. Importantly, however, IL-6 acts as a pyrogen in the presence of infection or inflammation. Thus, being a cytokine, it presents with a very short half-life and may decrease rapidly until the moment of clinical presentation [31]. Furthermore, cytokines usually lack specificity and are raised in a variety of inflammatory syndromes [42,70]. Overexpression of IL-6 is associated with a big range of pathological conditions, including multiple myeloma, Castleman’s disease, and rheumatoid arthritis [71].

## 7. Complete Blood Counts and Platelets

Complete blood counts are an easy, inexpensive, and routine examination technique that provides information about the composition of blood cells. Various types and ratios of blood cells, including neutrophil, platelet, lymphocyte, monocyte, neutrophil-to-lymphocyte ratio (NLR), platelet-to-lymphocyte ratio (PLR), and monocyte-to-lymphocyte ratio (MLR), have been proposed as indicators of systemic inflammation and infection [72,73]. Unfortunately, very few studies so far have investigated the diagnostic value of blood parameters in CAP. In those studies, NLR has been proven to be a promising candidate predictor of mortality in CAP patients [74]. On the other hand, MLR has also been recommended as a new indicator of disease severity in many diseases, such as rheumatic disease and cancer [75,76,77].

In a recent study involving 80 patients with CAP and 49 healthy individuals, it was demonstrated that NLR and MLR were both elevated in the patient group and had a higher diagnostic value for CAP compared to other blood parameters [15]. In the same study, NLR was shown to have a significant correlation with PSI and monocytes had a high diagnostic value for liver injury in CAP patients, overall indicating that NLR and monocyte levels may be reliable and cost-effective potential biomarkers for the diagnosis and severity evaluation of CAP [15]. In another study, Kartal et al. [74] reported that NLR and PLR are significantly increased in CAP and proposed that they can both be used as a predictor for the presence of CAP; however, they are not good inflammatory biomarkers for impatient and outpatient distinction.

Platelets are another possible biomarker for CAP severity as they play a very crucial role in the antimicrobial host defenses and the coagulation mechanism [78]. Thrombocytopenia and thrombocytosis have been significantly associated with mortality in CAP patients, and when compared to abnormalities in white blood cell counts, abnormalities in platelet counts are a better predictor of the severity and outcome of CAP [79].

## 8. Neutrophil CD64 Receptor

CD64 receptor is the high-affinity immunoglobulin Fc γ receptor on neutrophils (nCD64), and its function is to allow the opsonization and phagocytosis of microorganisms by these cells [80]. nCD64 increases during the proinflammatory state in response to infection and returns to normal when the stimulating factors disappear [80]. nCD64 expression is relatively stable in blood samples for more than 30 h; its detection requires a small blood volume; and the assay method for its detection is accurate, fast, and simple [81,82]. Several studies have investigated its role in the diagnosis of bacterial infection and sepsis [83,84,85,86,87,88,89,90]. With respect to CAP, in a prospective study on the expression of nCD64 determined by flow cytometry in blood samples from 83 adults with CAP, Burgos et al. [91] reported that patients with nCD64 expression greater or equal to 2700 mean fluorescent intensity (MFI) had more clinical deteriorations (36.4 vs. 7.2%, *p* = 0.015) and more ICU admissions (45.5 vs. 14.5%, *p* = 0.028), with a modest sensitivity of 44.4% for clinical deterioration and 33.3% for ICU admission and specificity of 90.1% and 90.8%, respectively. More studies are needed to delineate the value of neutrophil CD64 receptor in CAP.

## 9. Monocyte Human Leukocyte Antigen-DR (mHLA-DR)

Antigen-presenting cells display antigens to lymphocytes in association with major histocompatibility complex II molecules, such as the monocyte human leukocyte antigen-DR (mHLA-DR). This is crucial in order to develop sustained and adapted immune responses to clear the pathogen [92]. Measurement of HLA-DR expression on circulating monocytes is a rapid, inexpensive, and reproducible technique [93]. In sepsis, the mHLA-DR decreases rapidly in correlation to the severity and outcome of the septic shock [94,95]. Zhuang et al. [96] studied the expression of mHLA-DR by cytometry 24 h after admission to predict the 28-day survival of CAP patients. They reported that nonsurvivors significantly expressed reduced levels of mHLA-DR on their monocyte membranes [96]. There is still not enough evidence whether mHLA-DR could be a useful marker in CAP.

## 10. Presepsin

Presepsin is a fragment of monocyte lipopolysaccharide (LPS) receptor CD14 that is released in the blood during the process of bacterial phagocytosis. High levels of presepsin seem to predict the progression to septic shock and severe CAP, increasing the predictive and diagnostic accuracy of other markers, such as PCT, when measured in a combined assessment [97].

## 11. D-Dimer

D-dimer is a product of fibrin degradation and a widely used, easily measured biomarker for thromboembolic disease [31]. Blood levels of D-dimer reflect the pathological role of coagulation and fibrinolysis in the development of acute lung injury [31]. With respect to CAP, it has been reported that D-dimer levels are increased in patients with severe CAP and are associated with high risk of mortality [98], even in cases of CAP patients who do not present with an accompanying disease that could normally cause such a D-dimer increase [99]. Additionally, a serum D-dimer level <500 ng/mL on admission has been associated with a lower risk of death and morbidity in CAP, and it can therefore identify low-risk patients with CAP [100]. However, the coexistence of CAP and pulmonary embolism (PE) should be considered as elevated D-dimer levels significantly overlap between CAP and PE [101,102].

## 12. Triggering Receptor Expressed on Myeloid Cells 1 (TREM-1)

Triggering receptor expressed on myeloid cells 1 (TREM-1) is a glycoprotein member of the immunoglobin family [16]. Its expression is upregulated in the presence of extracellular bacteria and fungi and some noninfectious inflammatory conditions [16]. TREM-1 can be measured in body fluids only in response to infection as TREM-1 levels are not detectable in healthy individuals [16]. Grover et al. [103] found that TREM-1 is a good predictor of VAP; however, Palazzo et al. [104] claimed that TREM-1 can be found elevated in the bronchoalveolar lavage (BAL) fluid of patients with and without confirmed VAP. Therefore, further studies are required to fully determine the diagnostic power of TREM-1 for VAP.

## 13. Prohormones

Several prohormones, such as adrenomedullin (ADM), atrial natriuretic peptide (ANP), and arginine vasopressin (AVP) have also been associated with CAP.

ADM is produced during inflammation and is a potent vasodilator and bacteriocidal hormone with immunoregulatory effects [9]. Pro-ADM is a stable mid-regional fragment that is produced after degradation of ADM [9]. Pro-ADM has also been found to be increased in hypoxemia and to have an anti-inflammatory effect on bronchial epithelial cells and airway smooth muscle [105]. Other studies have shown that pro-ADM is particularly elevated in sepsis [106,107,108,109,110,111]. With respect to CAP, it has been shown that pro-ADM kinetics within the first 48 h after antibiotic administration can predict hospital mortality and has been proposed as a useful tool for assessment of CAP severity within the first hours [105].

ANP is a family member of the natriuretic peptides. Its biological role encompasses natriuresis, vasodilatation, dieresis, and inhibition of the renin–angiotensin–aldosterone axis and the sympathetic nervous system [112,113,114]. During sepsis, ANP levels are thought to increase rapidly, mainly in response to proinflammatory factors, such as IL-6 [115,116,117,118]. MR-pro-ANP is the mid-regional fragment of the precursor ANP hormone (pro-ANP) and is released in an equimolar ratio to ANP. As it is more stable than ANP in blood ex vivo, it is considered to be more applicable in clinical practice [119].

AVP is one of the key hormones of water homeostasis. Copeptin (or pro-AVP) is a precursor of arginine vasopressin and is raised in a variety of diseases, including congestive heart failure [31]. The C-terminal portion of copeptin (CT-pro-AVP) is cosecreted with AVP and is much easier to be quantified as AVP often binds to platelets and is unstable in isolated plasma [120,121]. It has been reported that the levels of CT-pro-AVP correlate significantly with poor outcomes in CAP sepsis [122,123,124,125]. Pro-ADM and copeptin have both been shown to be strong predictors of early mortality and adverse outcomes, potentially more than PCT and CRP [31]. Additionally, copeptin has been shown to be a useful marker of early mortality or ICU admission, more so than other biomarkers [124].

In a cohort study of 385 patients, elevated levels of ADM, AVP, and ANP were all associated with increased risk of death in patients with stable chronic obstructive pulmonary disease (COPD) [126]. In another study, daily walking activity was found as a predictor of circulating MR-pro-ANP and pro-ADM levels in stable COPD patients [127]. Thus, activity monitors can be used to stratify the risk of cardiac distress associated with long-term survival upon COPD exacerbation [127].

In a multivariate Cox proportional hazard regression analyses of 589 patients, high levels of MR-pro-ANP and CT-pro-AVP were compared to PCT and CRP and shown to be strongest predictors of mortality [128]. They were also found to be better predictors than the CRB-65 score for CAP severity and 28-day mortality [37]. Additionally, in a meta-analysis of eight studies and 4119 patients, MR-pro-ADM was proven to be predictive of increased complications and higher mortality rates in CAP patients as an elevated MR-pro-ADM level was associated with increased risk of death from CAP (relative risk = 6.16; 95% confidence interval = 4.71–8.06) [129]. Another study indicated that both hyponatremia and hypernatremia are significant predictors of 28-day mortality from CAP and that sodium levels increased the predictive potential of CT-pro-AVP and MR-pro-ANP [130].

## 14. Precision and Personalized Medicine

Recent investigations in CAP prognosis have brought into light the importance of individual characteristics. That is to say, various characteristics of the host are very important for the management and progression of pneumonia. Such characteristics include early clinical stability, the host’s inflammatory response and the response to antibiotics, the host’s susceptibility to specific organisms, the host’s genome and metabolic condition, and the patterns of the saprophytic flora colonizing the lower airways of the host [9].

## 15. Early Clinical Stability

Early clinical stability (normalization of heart rate, blood pressure, and rise of spontaneous O2 saturation >90% at day 3 after admission) has been associated with younger age, lower number of comorbidities, reduced symptoms and signs, and low platelet count. Early clinical stability is also associated with a significant lower 30-day and 90-day mortality rate, fewer ICU admissions, and shorter length of stay [131].

## 16. Metabolomics

Severe inflammatory processes cause many changes in cellular metabolism [9]. Neugebauer et al. [132] determined the levels of several metabolites, such as acylcarnitines, sphingolipids, and glycerophospholipids, during numerous inflammatory illnesses. They reported diverse metabolic patterns (metabolomes) specific for sepsis and CAP; moreover, they showed that putrescine (a polyamine) is a predictor for CAP [132]. Another study suggested that metabolomics are able to differentiate CAP from other noninfective pulmonary acute disorders with high specificity and sensitivity and that specific metabolites can significantly discriminate fatal from nonfatal CAP cases, thereby being predictors of survival [133]. Liu et al. [134] reported that thyroidal impairment during pneumonia seems to have a high predictive value. They observed that low T3 syndrome (characterized by low serum level of free triiodothyronine and normal to low level of thyroxine and thyroid-stimulating hormone) at 24 h after admission for CAP was associated with a higher rate of ICU admission and increased 30-day mortality [134].

Rapid detection of infectious diseases can be achieved by the exhaled breath, which contains volatile organic compounds (VOCs) that result from bacterial metabolism and/or host response to the environment [135]. Capture and analysis of VOCs in the exhaled breath has been shown to be safe and reliable in critically ill patients who are mechanically ventilated [136]. The presence of bacteria may be detected based on a small panel of VOCs [137].

## 17. Genomics

Genome-wide transcriptional studies have recently emerged as a powerful investigational tool. Personal genetic predisposition involved in response to a severe infection is also of great importance for the progression of pneumonia [9]. A genome-wide association study identified genetic variants that influence septic CAP outcome, with 11 loci significantly associated with 28-day survival of ICU patients admitted for severe septic CAP. One of these loci, the *FER* gene on chromosome 5, was found to have a single nucleotide polymorphism, resulting in a minor allele variant strongly associated with CAP survival (10% mortality among homozygotes for the alternative allele, 15% among the heterozygotes versus 25% in wild-type homozygotes) [138].

Sweeney et al. [139] identified an 11-gene set that was able to distinguish an infection from an inflammatory process and a seven-gene set to discriminate a viral or a bacterial cause of infection. The use of these two gene sets had a very high positive predictive value for the identification of a bacterial origin, with potential avoidance of antibiotic abuse [139].

Kothari et al. [140] reported that overexpression of the *TNFα* gene is associated with an increase in the incidence of severe sepsis and septic shock from all causes, including pneumonia. The *PIK3R3* gene that encodes the phosphoinositide 3 kinase regulatory subunit gamma, which is expressed in immune cells and is involved in chemoattractant-induced cell migration, has also been shown to contribute to sepsis and organ damage in critically ill patients [141]. None of these genes alone, however, can sufficiently enough indicate why certain patients develop VAP while others do not [16].

## 18. Microbiomics

An investigation of upper and lower respiratory microbiomes identified specific taxa differentially abundant in sputum samples of pediatric patients with CAP, which were subsequently stratified into different severity groups [142]. The sequencing of the bacterial 16S ribosomal RNA on respiratory specimens 24–48 h after admission led to the identification of specific patterns of lower airway microbiomes that were differently predictive of ICU admission and length of stay [142].

## 19. Proteomics

Proteomics applies the techniques of molecular biology, biochemistry, and genetics to analyze the structure, function, and interactions of the proteins produced by the genes of a particular cell or tissue [143]. A number of individual proteins have been proposed as biomarkers for the presence of VAP, but single biochemical measurements cannot be consistent predictors of either the presence or the severity of VAP [16]. Multibiomarker protein models could be more promising for the risk assessment of the disease [103,144,145]. Lu et al. [146] first described the BAL proteome from patients with VAP by identifying 206 proteins and creating a proteome map. Four of these 206 proteins—gelsolin, serum amyloid P-component, vitamin D-binding protein, and pyruvate kinase—were significantly higher in BAL from patients with VAP (*p* < 0.05). In addition, Nguyen et al. [147] identified a BAL protein signature (S100A8, lactotransferrin, actinin-1) that discriminates VAP patients with acute lung injury.

## 20. Conclusions and Future Perspectives

In severe pneumonia, the inflammatory response is complex and poorly controlled [148]. In such responses, it is necessary to deliver early, targeted, and effective treatment and to continually assess the patient’s clinical response while being aware of the advantages and limitations of all available clinical tools [31]. As the research on immune-modulatory therapies intensifies, so will the search for appropriate biomarkers for severity and treatment response [149]. Although our insight has significantly increased over the last years (Table 1), a translational approach with the application of genomics, metabolomics, and proteomics is required to better understand the disease. In this review, we discussed this rapidly evolving area and summarized the new era of novel biomarkers (Table 1 and Table 2). Diagnostic and predictive value is likely to increase only after the combination of scores and biomarkers or even by utilizing a biomarker panel, particularly in severely ill patients [150]. In future, data from large multisite trials in a diverse cohort of patients should provide us with precious evidence about appropriate surrogates for the severity and treatment response in CAP [31]. Moreover, further studies concerning the diagnostic value of blood parameters, which appear to be promising and cost-effective biomarkers for the diagnosis and severity evaluation of CAP, should be conducted. Additionally, we should aim for profound interactions and productive cooperation among basic science researchers and clinicians to push further translational research approaches, which could provide us with new insights and possibilities on pneumonia diagnosis. Last but not least, recent advances in the fields of metabolomics, genomics, proteomics, and microbiomics could further allow us to stratify patients into groups of different severity as personalized medicine is the ideal tool for determining a more accurate clinical outcome [9].

## Figures and Tables

**Table 1:** Summary of diagnostic, prognostic, and antibiotic guidance biomarkers in pneumonia and their indications.

Diagnostic Biomarkers	Prognostic Biomarkers	Antibiotic Guidance Biomarkers
Procalcitonin (PCT) [32]	C-reactive protein (CRP) predicts the absence of severe complications [60]	PCT guidance significantly reduces initiation and duration of antibiotic therapy [51]
CRP indicates inflammation intensity [40,58]	Interleukin 6 (IL-6) predicts treatment failure and mortality [39]	
Neutrophil CD64 (nCD64) used for the diagnosis of bacterial infection and sepsis [83,84,85,86,87,88,89,90]	Neutrophil-to-lymphocyte ratio (NLR) predicts mortality [74]	
D-dimer levels increased in patients with severe community-acquired pneumonia (CAP) [98]	Monocyte-to-lymphocyte ratio (MLR) indicates disease severity [75,76,77]	
Triggering receptor expressed on myeloid cells 1 (TREM-1) is a good predictor of ventilator-associated pneumonia (VAP) [103]	Platelets indicate CAP severity [78] and predict mortality [79]	
Atrial natriuretic peptide (ANP) levels increase during sepsis [115,116,117,118]	Monocyte human leukocyte antigen-DR (mHLA-DR) decreases rapidly in correlation to the severity and outcome of septic shock [94,95]; nonsurvivors express reduced levels of mHLA-DR [96]	
Metabolomics used to differentiate CAP from other noninfective pulmonary acute disorders [133]	Presepsin predicts severe CAP and progression to septic shock [97]	
Overexpression of *TNFα* gene is associated with severe sepsis and septic shock [140]	D-dimer level <500 ng/mL on admission indicates a lower risk of death and morbidity [100]	
*PIK3R3* gene expression contributes to sepsis and organ damage in critically ill patients [141]	Pro-adrenomedullin (ADM) within the first 48 h after antibiotic administration predicts hospital mortality [105]	
Gelsolin, serum amyloid P-component, vitamin D-binding protein, and pyruvate kinase are higher in bronchoalveolar lavage (BAL) from patients with VAP [146]	C-terminal portion of copeptin (CT-pro-AVP) correlates with poor outcomes in CAP sepsis [122,123,124,125], predicts early mortality or intensive care unit (ICU) admission [124]	
S100A8, lactotransferrin, actinin-1 discriminate VAP patients with acute lung injury [147]	Specific metabolites as predictors of survival [133]	
	Low T3 syndrome at 24 h after admission is associated with increased rate of ICU admission and increased 30-day mortality [134]	
	*FER* gene single nucleotide polymorphism results in a minor allele variant strongly associated with CAP survival [138]	
	Specific patterns of lower airway microbiomes differently predict ICU admission and length of stay [142]	
	Multibiomarker protein models for the risk assessment of CAP [103,144,145]	

**Table 2:** Summary of the biomarkers that indicate a direct evidence of infection and those that indicate the host’s response to infection.

Biomarkers That Indicate Direct Evidence of Infection	Biomarkers That Determine the Host Response to Infection
Presepsin is released in the blood during phagocytosis [97]	PCT, identifiable within 2–3 h with peak at 6 h [32]
TREM-1 expression is upregulated in the presence of extracellular bacteria and fungi [16]	CRP, identifiable within 4–6 h with peak at 36–50 h [40,59]
Diverse metabolomes specific for sepsis and CAP; putrescine is a predictor for CAP [132]	IL-6, immediate response to infection [44,69], more sensitive for localized infection (e.g., effusions) [44]
Exhaled breath contains volatile organic compounds (VOCs) that result from bacterial metabolism and/or host response to the environment [135]	NLR, PLR, and MLR indicate systemic inflammation and infection [72,73]
Specific patterns of lower airway microbiomes differently predict ICU admission and length of stay [142]	nCD64 increases during the proinflammatory state in response to infection and returns to normal when the stimulating factors disappear [80]
	Early clinical stability is associated with a significant lower 30-day and 90-day mortality rate, fewer ICU admissions, and shorter length of stay [131]
	Personal genetic predisposition is involved in the response to a severe infection and predicts the progression of pneumonia [9]
	Individual proteins as biomarkers for the presence of VAP [16]
